# High Visual Working Memory Capacity in Trait Social Anxiety

**DOI:** 10.1371/journal.pone.0034244

**Published:** 2012-04-09

**Authors:** Jun Moriya, Yoshinori Sugiura

**Affiliations:** 1 Department of Experimental Clinical and Health Psychology, Ghent University, Ghent, Belgium; 2 Graduate School of Integrated Arts and Sciences, Hiroshima University, Hiroshima, Japan; Ghent University, Belgium

## Abstract

Working memory capacity is one of the most important cognitive functions influencing individual traits, such as attentional control, fluid intelligence, and also psychopathological traits. Previous research suggests that anxiety is associated with impaired cognitive function, and studies have shown low verbal working memory capacity in individuals with high trait anxiety. However, the relationship between trait anxiety and visual working memory capacity is still unclear. Considering that people allocate visual attention more widely to detect danger under threat, visual working memory capacity might be higher in anxious people. In the present study, we show that visual working memory capacity increases as trait social anxiety increases by using a change detection task. When the demand to inhibit distractors increased, however, high visual working memory capacity diminished in individuals with social anxiety, and instead, impaired filtering of distractors was predicted by trait social anxiety. State anxiety was not correlated with visual working memory capacity. These results indicate that socially anxious people could potentially hold a large amount of information in working memory. However, because of an impaired cognitive function, they could not inhibit goal-irrelevant distractors and their performance decreased under highly demanding conditions.

## Introduction

Working memory enables people to maintain task-relevant information in a highly active state. Although everyday life is filled with a great deal of visual information, our visual working memory can maintain representations of only three to four objects at a time [1], [2], [3], [4], [5], [6]. However, visual working memory capacity is not necessarily constant, but varies across individuals [4], [7], [8], [9]. Different working memory capacities among individuals affect several cognitive abilities, such as fluid intelligence [10], [11], [12] or stereotype [13], [14]. Working memory performance is also severely disrupted in people with psychopathological traits, such as anxiety.

Anxiety consumes the working memory capacity available for superior performance [15], [16]. Depleted working memory capacity during worry is especially observed in highly anxious people [17], [18]. Considering this, many researchers believe that anxious people have diminished working memory capacity because anxiety occupies their working memory. In fact, some studies have shown that reduced working memory capacity is associated with anxiety [19], [20]. Amir and Bomyea [19] used the operation span paradigm (OSPAN) [21], in which participants remember sequentially presented words with simple math equations. They have shown that individuals with social anxiety disorders remember fewer words than non-anxious individuals do. However, few previous studies have investigated visuospatial working memory. Considering that the important role of working memory is to maintain representations spatially and simultaneously [1], [2], [3], [4], [5], [6], it is necessary to examine the visual working memory capacity at one time in anxiety.

In attentional control theory [22], Eysenck mentioned that when people perceive themselves to be under threat and experience anxiety, it is advantageous to allocate visual attention more widely in order to detect threatening stimuli. Based on this hypothesis, it is possible that individuals with anxiety attend to and hold many stimuli at a time. Actually, previous studies have shown the possibility of high attentional resources, but not visual working memory capacity, in high trait anxiety [23], [24], [25], [26]. Bishop [23] used a perceptual load task [27], [28] in which a target was present in the center and a task-irrelevant distractor was present in the peripheral field. The stimuli were not emotionally laden and consisted of simple letters. Participants were instructed to detect the target without processing the task-irrelevant distractor. However, individuals with high trait anxiety, but not state anxiety, detected both the target and the task-irrelevant distractors. According to the perceptual load theory [28], attention has a limited capacity and the processing of task-irrelevant distractors depends on attentional resources. Individuals who had few attentional resources devoted all of them to the targets and could not detect task-irrelevant stimuli. However, individuals who have sufficient attentional resources can devote some to the target, but spare resources remain. The remaining resources are allocated to peripheral task-irrelevant stimuli. Therefore, it is possible that the processing of task-irrelevant distractors in individuals with trait anxiety was observed because they have more attentional resources than those with low trait anxiety. Considering that visual working memory and visual attention are intimately related [29], [30], [31], we assume that individuals with high trait anxiety also have high working memory capacity compared to those with low trait anxiety.

However, the processing of task-irrelevant distractors in high trait anxiety in previous studies also indicates the possibility that individuals with high trait anxiety have low visual working memory capacity. Based on a recent cognitive model in anxiety [32], top-down control, which includes inhibition of task-irrelevant stimuli, is impaired in trait anxiety but not in state anxiety. It is possible that individuals with high trait anxiety could not inhibit the task-irrelevant distractors because of impaired top-down control. Therefore, the processing of task-irrelevant distractors was observed with high trait anxiety. In fact, individuals with high trait anxiety showed reduced activity in the prefrontal cortex, which is associated with top-down control [23]. This relationship between high trait anxiety and low top-down control was observed even when controlling for state anxiety. Because the role of working memory is to maintain task-relevant information and ignore task-irrelevant stimuli, these previous studies predict that even with visual information, trait anxiety, but not state anxiety, might be associated with low working memory and low visual working memory capacity. However, these previous studies investigated attention, and the relationship between anxiety and visual working memory capacity is not yet clear.

The present study investigated the effects of social anxiety on visual working memory capacity. We focused on trait social anxiety, which is related to anxiety in social situations (e.g., meeting new people, public speaking, and going to parties) and fear of negative evaluation from others [33], [34]. Individuals with social anxiety are too sensitive to evaluation from others and fearful of negative evaluation [35]. They are very vigilant to social information in social situations, such as facial expressions and gaze direction of others [36], [37], [38]. A recent study also showed that individuals with social anxiety are not only sensitive to social or emotional stimuli but also to non-emotional visual stimuli, such as bright stimuli [39]. High sensitivity to visual stimuli might be due to fear that is activated by visual images in socially anxious individuals [40]. General anxiety, however, consists mainly of thought and is not necessarily associated with concrete visual images [41], [42]. Therefore, we assume that visual working memory is more associated with trait social anxiety. We also measured the degree of state anxiety. According to recent studies and models [23], [24], [25], [26], [32] that show impaired top-down attention and processing of task-irrelevant stimuli, trait anxiety, but not state anxiety, might be associated with visual working memory capacity. We used a change-detection task in this study, a method that is commonly used and is an established task for measuring visual working memory capacity [3], [8]. In this task, participants are shown an array of visual stimuli to encode. A test array is presented after a short retention interval, and participants are required to answer whether the test array is identical to or different from the memory array. The accuracy of the task is used to estimate visual working memory capacity [43], [44]. Based on the high attentional resources and the wide visual attentional allocation in trait anxiety [22], visual working memory capacity might be positively correlated with trait social anxiety but not state anxiety. However, according to the theory of impaired top-down control in trait anxiety, visual working memory might be negatively correlated with trait social anxiety but not state anxiety.

## Results

### Experiment 1

**Figure 1:**
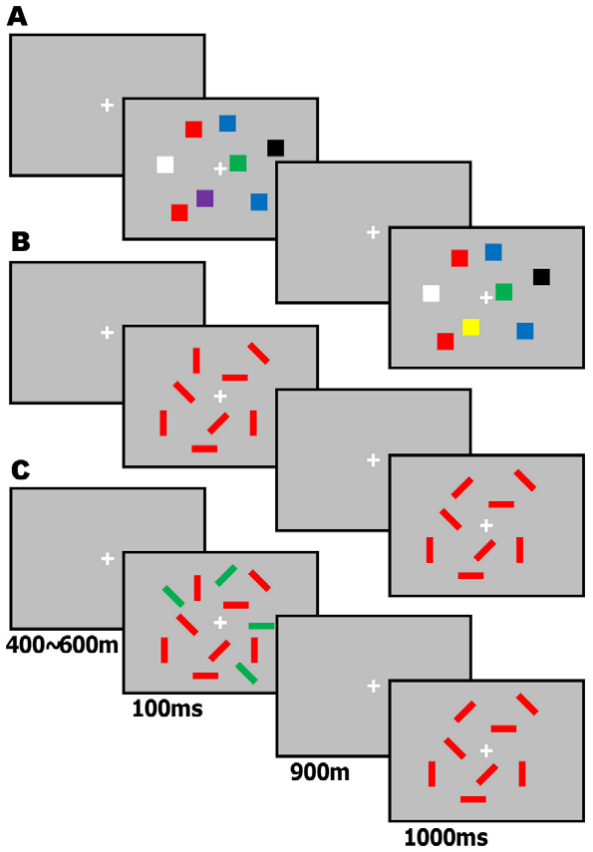
Experimental procedure. **A.** Example of a visual memory trial in Experiment 1, wherein the color stimulus between memory and test arrays is different. **B.** Example of a visual memory trial in Experiment 2, wherein the orienting stimulus between memory and test arrays is different. **C.** Example of a visual memory trial under target with distractor conditions in Experiment 3. Time scales in the three experiments are all the same.

On each trial, a memory array of 4, 8, or 12 colored squares was presented for 100 ms, and participants were asked to remember the items (Fig. 1A). Memory was tested 1 s later with a test array that was either identical to the memory array or differed by one color, and participants were required to indicate whether the two arrays were identical or different. We estimated each individual's visual working memory capacity (K) by averaged capacities for set-sizes 8 and 12 [43].

**Figure 2:**
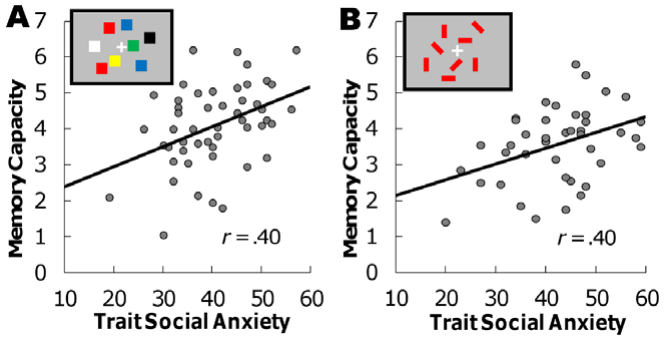
Results from Experiments 1 and 2. **A.** The correlation between trait social anxiety scores and individual visual working memory capacity in Experiment 1. **B.** The correlation between social anxiety and memory capacity in Experiment 2.

We measured the correlations among trait social anxiety, state anxiety, and memory capacity. A positive correlation between trait social anxiety and memory capacity was found to be significant (*r* = .40, *p*<.01, Fig. 2A), whereas state anxiety was not correlated with memory capacity (*r* = .24, *ns*). A multiple regression analysis predicting memory capacity from trait social anxiety and state anxiety showed a significant model (*F* (2, 47) = 5.33, *p*<.01, *R^2^* = .19). Trait social anxiety was a statistically significant predictor (*β* = .37, *p*<.01); however, state anxiety was not (*β* = .15, *ns*).

These results support the hypothesis of high attentional resources in trait anxiety [22]. No studies have demonstrated high visual working memory capacity in social anxiety. In Experiment 2, we confirmed this phenomenon and examined whether this relationship would be applied to other feature dimensions.

### Experiment 2

On each trial, a memory array of red rectangles with varied orientations was presented, and participants were asked to remember the orientations of the items (Fig. 1B). Memory for the items was tested 1 s later with a test array that was either identical to the original memory array or differed by one orientation.

The results showed that trait social anxiety was positively correlated with memory capacity (*r* = .40, *p*<.01, Fig. 2B), whereas state anxiety was not (*r* = .01, *ns*). A multiple regression analysis showed a significant model (*F* (2, 38) = 3.64, *p*<.05, *R^2^* = .16). Trait social anxiety was a statistically significant predictor (*β* = .41, *p*<.01); however, state anxiety was not (*β* = −.06, *ns*).

Trait social anxiety was positively correlated with visual working memory capacity. These results also support the hypothesis that individuals with trait social anxiety have high attentional resources. However, it is still unclear whether socially anxious people maintain high working memory capacity when presenting task-irrelevant distractors like previous studies [23], [24], [25], [26]. If impaired inhibition of task-irrelevant stimuli occurs in high trait social anxiety, individuals with social anxiety would not inhibit the distractors and would allocate their high working memory resources to them. Consequently, socially anxious people could not sufficiently allocate attention to task-relevant stimuli; therefore, high visual working memory capacity for task-relevant stimuli might not be observed in those with social anxiety. In Experiment 3, we showed not only targets but also task-irrelevant distractors.

### Experiment 3

The procedure was identical to Experiment 2 with the following modification: on half of the trials, four green rectangles were also present (Fig. 1C). Participants were required to remember the orientations only of the red items. We estimated not only memory capacity but also filtering efficiency [45]. Filtering efficiency refers to the degree to which performance under distracting conditions is similar to performance with targets only and shows how efficiently participants filtered out the distractors.

**Figure 3:**
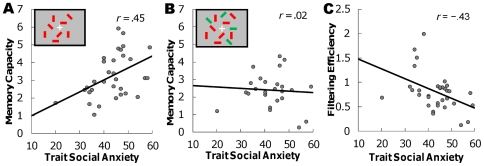
Results from Experiment 3. **A.** The correlation between trait social anxiety scores and individual visual working memory capacity under no-distractor conditions. **B.** The correlation between social anxiety and memory capacity under target with distractor conditions. **C.** The correlation between social anxiety and filtering efficiencies.

The results showed that under no-distractor conditions, which are the same as those in Experiment 2, trait social anxiety was positively correlated with memory capacity (*r* = .45, *p*<.01, Fig. 3A), whereas state anxiety was not correlated with memory capacity (*r* = .10, *ns*). A multiple regression analysis showed a significant model (*F* (2, 30) = 3.81, *p*<.05, *R^2^* = .20). Trait social anxiety was a statistically significant predictor (*β* = .46, *p*<.05), but state anxiety was not (*β* = −.04, *ns*). However, under distractor conditions, in which there were task-irrelevant distractors, memory capacity was not correlated with either trait social anxiety (*r* = .02, *ns*, Fig. 3B) or state anxiety (*r* = −.23, *ns*). These results might reflect impaired filtering efficiency. Filtering efficiency was negatively correlated with trait social anxiety (*r* = −.43, *p*<.05, Fig. 3C) and with state anxiety (*r* = −.37, *p*<.05). A multiple regression analysis showed a significant model (*F* (2, 30) = 4.81, *p*<.05, *R^2^* = .24). Trait social anxiety was a statistically significant predictor (*β* = −.35, *p*<.05), but state anxiety was not (*β* = −.26, *ns*).

We applied the Smirnov-Grubbs analysis and found one outlier, which scored 20 on the BFNE. We excluded this participant and reanalyzed the data. The results are identical to above-mentioned results. Under no-distractor conditions (*F* (2, 29) = 2.80, *p* = .077, *R^2^* = .16), trait social anxiety predicts significantly high visual working memory capacity (*β* = .41, *p*<.01), whereas state anxiety does not predict memory capacity (*β* = −.04, *ns*). However, under distractor conditions (*F* (2, 29) = 1.21, *ns*, *R^2^* = .08), trait social anxiety was not associated with visual working memory capacity. Filtering efficiency was predicted from trait social anxiety (*β* = −.46, *p*<.01), but not state anxiety (*β* = −.26, *ns*), when conducting multiple regression analysis (*F* (2, 29) = 7.45, *p*<.01, *R^2^* = .34).

Again, trait social anxiety was positively correlated with visual working memory capacity under the no-distractor condition. Individuals with high trait social anxiety have potentially high visual working memory capacity. However, this correlation was not observed when the task-irrelevant distractors were present. Considering the negative correlation between social anxiety and filtering efficiency, socially anxious people could not filter out the distractors because of impaired top-down attention, and their resources were allocated to both task-relevant and task-irrelevant stimuli. Therefore, visual working memory capacity for task-relevant stimuli might not increase as trait social anxiety increases under the distractor condition.

In all experiments, the performance of some participants is close to chance level. We conducted a one-sample t-test for each participant to reveal whether their performance was distinguishable from chance level (i.e., 0.5 in correct rates). Based on the analysis, we excluded 2, 3, and 2 participants in Experiment 1, 2, and 3, respectively. We reanalyzed the data and the results were almost identical to those with all participants. Trait social anxiety was positively correlated with visual working memory capacity (Experiment 1: *r* = .40, *p*<.01; Experiment 2: *r* = .33, *p*<.05; Experiment 3: *r* = .49, *p*<.01), but state anxiety was not correlated with memory capacity (Experiment 1: *r* = .23, *ns*; Experiment 2: *r* = −.08, *ns*; Experiment 3: *r* = .09, *ns*). In Experiment 3, filtering efficiency was negatively correlated with trait social anxiety (*r* = −.39, *p*<.01) and marginally significant with state anxiety (*r* = −.33, *p* = .068). Therefore, we do not think that the present results are dependent on response bias.

We also measured trait anxiety with the STAI-Trait Form (STAI-T) in all experiments. The results of correlations between working memory capacity and trait anxiety were almost the same as those with trait social anxiety. Except in experiment 2, trait anxiety was positively correlated with visual working memory capacity (Experiment 1: *r* = .27, *p* = .054; Experiment 2: *r* = .20, *ns*; Experiment 3: *r* = .48, *p*<.001). In addition, trait anxiety was negatively correlated with filtering efficiency in Experiment 3 (*r* = −.37, *p*<.05). Therefore, we could not assert that high visual working memory capacity applies only to trait social anxiety. However, we assume the high visual working memory capacity might be highly influenced by social anxiety.

## Discussion

Previous studies have shown that reduced working memory capacity for verbal stimuli or sequential visual stimuli is associated with anxiety [19], [20]. However, few studies have investigated visual working memory capacity at one time in anxiety. The present study shows that socially anxious people have a high visual working memory capacity. At the same time, they had difficulty ignoring task-irrelevant distractors because of impaired top-down control. When presented with task-irrelevant distractors, they allocated resources to them, and high visual working memory capacity was not observed. However, because they have sufficient visual working memory capacity, they could allocate enough resources to task-relevant targets comparable to individuals with low trait social anxiety even with the presence of task-irrelevant distractors. Socially anxious people might widely allocate their working memory resources, and as a result, they hold both non-threatening and threatening stimuli simultaneously. In public speaking, for example, socially anxious people might hold the reactions of many audience members, some of whom might show negative reactions. Even though their goal is not to direct attention to audience members but to give a fluent talk, socially anxious people might not filter out the reactions of the audience.

Although high visual working memory capacity had an effect on filtering efficiency in previous studies [7], [9], [46], the results in the present study were inconsistent with these findings. Individuals with high working memory capacity generally have good skills for cognitive tasks; however, their performance is impaired in certain conditions, such as under high pressure [15], [47], [48]. Trait social anxiety might play a role that is similar to pressure. Individuals with social anxiety are too sensitive to evaluation from others and are afraid of a negative evaluation [35]. This fear of negative evaluation might create a high-pressure situation. Social anxiety moderates the relationship between high memory capacity and cognitive skills and decreases filtering efficiency. Socially anxious people try to maintain high performance or effectiveness, which refers to an individual's competence in doing a task [22], [49]. Moreover, considering the positive relationship between high visual working memory capacity and fluid intelligence [10], [11], [12], socially anxious people might potentially have higher cognitive ability. However, excessive fear of negative evaluation from others leads to high pressure; consequently, socially anxious people showed decreased filtering performance.

While the present study provides important findings for high visual working memory capacity in trait social anxiety, we are aware of some limitations. First, we measured state anxiety but did not manipulate it. Some previous studies investigated the role of state anxiety by inducing negative mood, such as presenting negative stimuli before the task or threat of electric shock [50], [51], [52]. Further studies need to manipulate state anxiety directly to reveal the effects of state anxiety on visual working memory capacity. Second, the present study examined non-clinical individuals. It is not clear whether the present results apply to clinically diagnosed individuals, such as those with social anxiety disorders. The previous studies have shown that the average Brief Fear of Negative Evaluation Scale (BFNE) score in people suffering from social anxiety disorders is 49.3 [53]. In the present study, the average BFNE score (and standard deviation) was 41.8 (8.7). About 17% of the participants in the present study scored above 49, and this is a considerable number. It is possible that high visual working memory capacity is observed in clinical samples. If so, further studies should confirm this. Third, we could not reveal the relationship between high visual working memory capacity and threat detection. High visual working memory capacity might enable highly anxious people to detect a threatening stimulus among many stimuli. In addition, individuals with social anxiety hold socially threatening stimuli efficiently [19]. Further study should investigate visual working memory capacity with threatening stimuli in anxious individuals. Finally, we could not exclude the effects of variables other than trait social anxiety and state anxiety, such as intrinsic motivation or IQ. In particular, fluid intelligence is positively associated with working memory capacity [10], [11], [12]. Further study should investigate the interaction between these factors and social anxiety on visual working memory capacity.

In summary, the present study investigated the relationship among trait social anxiety, state anxiety, and visual working memory capacity. These results provide the first evidence of a relationship between high visual working memory capacity and high social anxiety and show the importance of considering individual differences in visual working memory capacity in psychopathological traits. Although working memory training to increase capacity is now used in several areas [54], [55], [56], the present results indicate that it is important to elucidate which aspects of working memory should be targeted in the treatment of anxiety.

## Methods

### Participants

Fifty university students (19 males and 31 females, age range of 18–27) participated in Experiment 1, 41 university students (10 males and 31 females, age range of 18–22) participated in Experiment 2, and 33 university students (8 males and 25 females, age range of 18–22) participated in Experiment 3. They were required to complete a written informed consent form before participating in the study. Before the experiments, we asked participants whether they could detect colored stimuli and ascertained that they could do so. After the experiments, participants completed questionnaires. The institutional review board and ethics committee in Hiroshima University approved our study. Cash was given in return for their participation.

### Questionnaires

The Brief Fear of Negative Evaluation Scale (BFNE): The BFNE assesses apprehension as a result of others' negative evaluations [35], [57]. It is a commonly used measure that reflects the degree of trait social anxiety. It comprises 12 items using a 5-point Likert scale. Internal consistency (Cronbach's alpha) was .92 and test-retest reliability was .74.

The State-Trait Anxiety Inventory-State Form (STAI-S) and Trait Form (STAI-T): The STAI-S measures state anxiety as a transitory emotional state characterized by subjective, consciously perceived feelings of tension and is commonly used to measure state anxiety while the STAI-T measures trait anxiety as a relatively stable personality trait [58], [59]. It comprises 20 items using a 4-point Likert scale. Internal consistency (Cronbach's alpha) was .87, and test-retest reliability was .80. There are no proper scales that measure state social anxiety at this time. Therefore, we used a state anxiety scale in the present study rather than measuring state social anxiety with other methods, such as a visual analog scale. The STAI-S has higher internal consistency and test-retest reliability compared with a visual analog scale.

### Stimuli and Procedure

All stimulus arrays in Experiments 1, 2, and 3 were presented within a 9.8°×7.3° region on a monitor with a gray background, and the items were separated by at least 2.0° (center to center). One feature of one item in the test array was different from the corresponding item in the sample array on 50% of trials; the sample and test arrays were otherwise identical. Participants were required to indicate whether the two arrays were identical or different.

In Experiment 1, we showed 4, 8, or 12 colored squares (0.65°×0.65°). Each square was selected at random from a set of seven highly discriminable colors (red, blue, violet, green, yellow, black, and white), and a given color could appear no more than twice within an array. Stimulus positions were randomized on each trial. There were 80 trials in each set size for a total of 240 trials.

In Experiment 2, we showed 4, 8, or 12 red rectangles (0.12°×0.52°) selected randomly from a set of four orientations (vertical, horizontal, left 45°, and right 45°). Stimulus positions were randomized on each trial. There were 80 trials in each set size for a total of 240 trials.

The method for Experiment 3 was identical to Experiment 2 except for the following modification: on half of the trials, there were four green distractors (0.12°×0.52°) selected randomly from a set of four orientations (vertical, horizontal, left 45°, and right 45°). Stimulus positions were randomized on each trial. There were 60 trials in each set size and the distractor condition for a total of 360 trials.

### Analysis

We estimated each individual's memory capacity with a standard formula [43]. The formula is K = S (H−F), where K is the memory capacity, S is the size of the array, H is the observed hit rate, and F is the false alarm rate. We also estimated the filtering efficiency in Experiment 3 according to a previous study [45]. Filtering efficiency was calculated as a ratio comparing the K score under target with distractor conditions to the K score under no-distractor conditions with the same set size of the targets. Filtering efficiency reflects the degree to which performance under distracting conditions is similar to performance with targets only. If a participant's performance is unaffected by the presence of distractors and is absolutely the same as the performance in no-distractor conditions, the filtering-efficiency score is 1. The more the distractors interfered with a participant's performance, the smaller the filtering efficiency score.

Considering that an average capacity of visual working memory is typically around three to four items, individual differences in memory capacity might not be observed with low set sizes of less than four items [3], [7], [8], [60]. In order to capture individual differences, we focused on the average K-estimates and filtering efficiency for set sizes 8 and 12.
